# The importance of corneal biomechanics in assessing first degree family members of keratoconus patients

**Published:** 2018

**Authors:** Ioana Catalina Ionescu, Catalina Gabriela Corbu, Cristina Nicula, Valeria Coviltir, Vasile Potop, Mihaela Constantin, Dana Dascalescu, Miruna Burcel, Veronica Strehaianu, Radu Ciuluvica, Liliana-Mary Voinea

**Affiliations:** * Clinical Ophthalmology Emergency Hospital, Bucharest, Romania; ** Oftaclinic Clinic, Bucharest, Romania; *** Department of Ophthalmology, Faculty of Medicine, “Carol Davila” University of Medicine and Pharmacy, Bucharest, Romania; **** Department of Ophthalmology, Faculty of Medicine, “Iuliu Hatieganu” University of Medicine and Pharmacy, Cluj-Napoca, Romania; ***** Clinical Emergency Hospital Cluj-Napoca, Cluj-Napoca, Romania; ****** Faculty of Dental Medicine, “Carol Davila” University of Medicine and Pharmacy, Bucharest, Romania; ******* Department of Ophthalmology, University Emergency Hospital, Bucharest, Romania

**Keywords:** keratoconus, biomechanics, relatives

## Abstract

**Aim:** to determine the values of biomechanical parameters (corneal hysteresis - CH, corneal resistance factor - CRF and keratoconus match index - KMI) in patients with keratoconus and their first degree family members. The purpose of the present study was to investigate the importance of assessing corneal biomechanics in subjects at risk of developing the primary ectasia.

**Materials and methods:** 48 participants divided into three groups were analyzed in an observational study after a complete ophthalmological exam with the primary focus on Ocular Response Analyzer.

**Results:** The mean values of CH, CRF, and KMI in the group of relatives were lower compared with the controls but higher when compared with keratoconus patients. We noted significant differences of CH and CRF between all three groups, while in the case of KMI, only the keratoconus group presented statistically significant differences compared with the relatives, respectively with the healthy subjects.

**Conclusions:** the decreased values of CH and CRF may raise the question whether corneal biomechanics could be an adjuvant tool in the screening of a first-degree family member of a keratoconus patient in the attempt of the early detection of a possible forme fruste keratoconus.

## Introduction

Keratoconus is a progressive, mostly bilateral disorder which results in the thinning of the corneal stroma, thus modifying its normal architecture into a conical shape [**1**,**2**]. 

This can lead to irregular astigmatism and corneal scarring with a significant impact on the visual acuity [**3**]. The disease starts to develop usually in the early puberty and continues to progress until approximately the fourth decade of life [**4**]. Described as a noninflammatory disease, the pathophysiology of keratoconus remains enigmatic. It is considered to be a multifactorial corneal ectasia triggered by external factors such as eye-rubbing or contact lens wear and endogenous stimuli through the interplay of inflammatory tear mediators, a dysregulation of oxidative stress and proteolytic enzymes resulting in corneal remodeling and keratocytes apoptosis, as well as the presence of atopic patient history [**5**-**8**]. Furthermore, the condition has also a genetic pathway, most cases being sporadic, isolated, or associated with other ocular or systemic diseases like Down, Marfan, Ehler-Danslos syndromes [**9**]. Many studies reported an important number of cases with familial inherited keratoconus, either through an autosomal dominant or recessive transmission, suggesting the higher risk in first-degree family members of developing the disease [**10**-**12**].

Classically, keratoconus was defined as a noninflammatory corneal condition, yet in the last decade, many authors have debated the possible role of inflammation. Recent studies have published results that highlight the cytokine overexpression in the tear film of keratoconus patients [**13**-**16**]. 

An essential parameter in monitoring the disease progression is the corneal biomechanics, which offers in vivo measurements of the cornea while being deformed when a mechanical stress is applied [**17**]. From a histopathological point of view, a keratoconic cornea has certain changes such as the thinning of the epithelium, the presence of breaks in Bowman’s layer, disorganized and reduced collagen fibrils that have repercussions on the corneal biomechanics [**18**]. The cornea is a viscoelastic material and exhibits the property of recovering the initial form after the applied stress with a lag between the application of the force and the response. This property is called hysteresis [**19**].

Given the fact that the corneal stroma and Bowman’s layer are responsible for the corneal strength, their disintegration may cause instability of the tissue. Proteolytic enzymes and inflammatory cytokines released in the tear film of keratoconus patients generate a corneal thinning through the alterations produced at the level of the extracellular matrix and collagen fibrils. These factors interfere in the mechanical stability and imbalance the viscous behavior of the cornea [**20**-**23**].

Multiple methods have been used to measure corneal biomechanics beginning with the ex vivo studies reflected by Young’s modulus and continuing with the in vivo assessment of biomechanical properties. The Ocular Response Analyzer (ORA; Reichert Ophthalmic Instruments, Buffalo, New York, USA) is a noncontact tonometer that indents the cornea through an air puff producing two distinct peaks: P1 moving inwards and P2 outwards, representing the necessary pressures to deform the cornea respectively to recover from the applanation while the air pulse decreases. The difference between P1 and P2 is an indicator of the corneal viscosity and is called hysteresis (CH). As for the other parameter, the corneal resistance factor (CRF) highlights the overall resistance and is referred to as the indicator of corneal elasticity [**24**-**26**]. In the last years, ORA has been used in the attempt of early detection of subclinical keratoconus and for monitoring its progression. According to many studies, both parameters (CH and CRF) have been reported to be lower in keratoconic corneas compared to healthy ones [**25**,**27**].

Another waveform analysis is represented by the keratoconus match index (KMI), which is believed to be a reliable index in keratoconus diagnosis and staging, especially in the early diagnosis of a subclinical keratoconus and results from the comparison of the waveform of the patient’s eye compared with the waveform of the normal population in the database [**28**-**30**].

## Patients and methods

This study included forty-eight patients divided into three groups: 17 eyes of 17 patients in the set of keratoconus, with a mean age of 23.35 years, 16 eyes of 16 first degree family members of the keratoconus patients, ranging from 9 to 30, with a mean age of 18.81 years, and the control group with 15 eyes, with a mean age of 28.66 years. All 48 participants were followed from January 2016 to July 2017. Prior to their enrollment, the patients have provided the written informed consent and the institutional ethics procedure was approved by the Ethics Committee of “Carol Davila” University of Medicine and Pharmacy, Bucharest. In order to recruit the participants, we performed a complete ophthalmic clinical and paraclinical examination including personal and family history taking, full clinical exam, corneal topography, Ocular Response Analyzer, pachymetry. Our present study concerns the evaluation of the three important biomechanical parameters assessed by ORA in the three groups: corneal hysteresis (CH), corneal resistance factor (CRF) and Keratoconus Match Index (KMI). Exclusion criteria were history of ocular surgery (corneal crosslinking, cataract surgery, refractive surgery that could affect the biomechanical characteristics), glaucoma, and ocular hypertension. As for the inclusion criteria, we recruited patients diagnosed with keratoconus using the Amsler Krumreich classification (based on topography, central corneal thickness, and refraction error). For the group of relatives and controls we chose the ones with normal topography indices and no clinical signs of keratoconus.

The statistical analysis was completed using SAS University Edition version 9.3 and R - version 3.4.0. The comparison between the already mentioned variables (CH, CRF, and KMI) of the three sets was made with an ANOVA test if the distribution of the variables could be approximated by a normal one, and if not, we chose to perform a Kruskal-Wallis test.

## Results

In the present study, the mean value of CRF in the set of keratoconus was 7.40 (±2.14), ranging from 3.70 to 12.10, while in the first degree family members’ group the value ranged from 7.20 to 13.60 with a mean CRF of 10.21 (±1.78) and the control group with a mean CRF of 12.95 (±1.79). We can state that the mean value of CRF was the lowest in the keratoconus group, followed by the relatives and the highest in the control group. 

Given the fact that the distribution of the variable was similar to the Gaussian distribution in all groups, we used the ANOVA test to see if the mean differences between the groups have statistical significance. We observed significant differences between the 3 pools and performed Tukey’s honest significant difference test to find which pairs were different from each other. There were statistically significant differences between all groups (p<0.01), with a confidence interval that did not include 0.

**Fig. 1:**
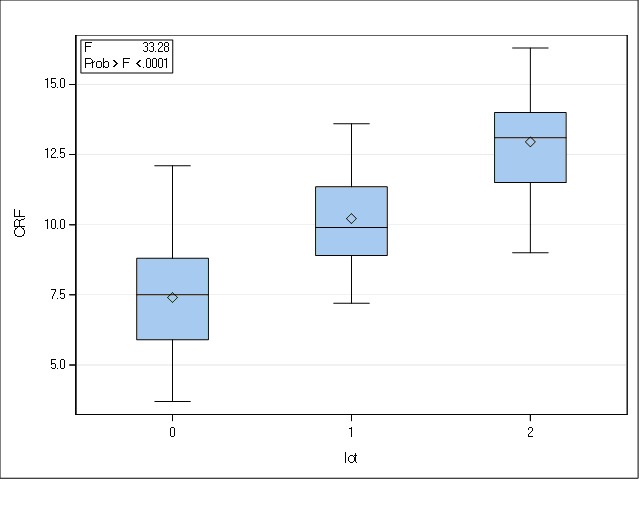
Distributions of CRF in the three groups (0 = keratoconus group; 1 = family members, 2 = controls)

**Table 1:** Statistically significant differences between the three pools

Contrast CRF	Mean differences	Tukey adjusted p value	CI95%
0 vs. 1	-2.81	0.0003	-4.43 to -1.19
0 vs. 2	-5.54	< 0.0001	-7.19 to -3.89
1 vs. 2	-2.73	0.0007	4.40 to -2.73

Further, we analyzed the corneal hysteresis and found similar results. The mean CH in the group of keratoconus patients was 8.27 (±1.97) with a range between 6.10 and 13.40, while the relatives had a mean value of 10.36 (±1.75) ranging from 8.20 to 13.30 compared to the control subjects that recorded a mean value of 13.05 (±1.41). CH, as well as CRF, was poorly represented in the keratoconus group, followed by the relatives, while the controls had the highest value. 

**Fig. 2:**
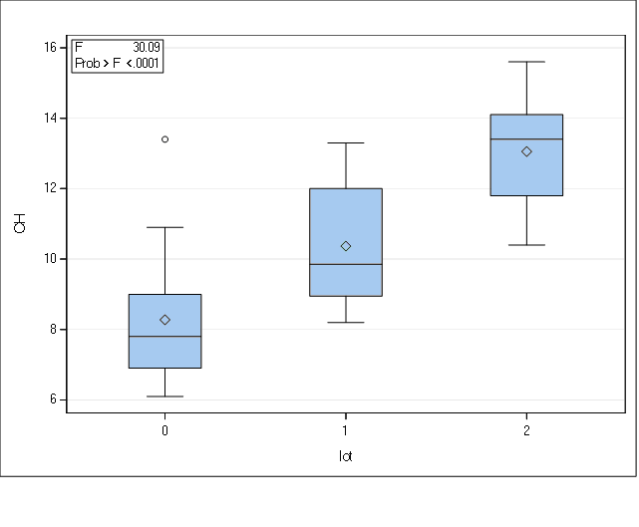
Distributions of CH in the three groups (0 = keratoconus group; 1 = family members, 2 = controls)

In the case of the keratoconus group, the distribution could not be approximated within the Gaussian distribution; hence, we performed a Kruskal-Wallis test. This result (χ² = 26.92, gl = 2, p < 0.01) and another test as a finishing point of this finding (a corrected post-hoc Kruskal-Wallis test-Dunn) suggested the existence of statistically significant differences between all three groups, as shown in the table below.

**Table 2:** Statistically significant differences between the three pools

Contrast CH	Adjusted p value
0 vs. 1	0.03
0 vs. 2	< 0.0001
1 vs. 2	0.01

The last parameter in evaluating corneal biomechanics was KMI, a codified score measured at each ORA analysis. In the group of keratoconus patients, we found the lowest score, with a mean of 0.07 (±0.22) ranging from -0.30 to 0.54, followed by a higher value of 0.69 (±0.15) in the group of relatives and culminating with the highest mean score of 0.78 (±0.16) in the control set.

The descriptive statistical analysis showed that the distribution of the variable was normal in all groups.

**Fig. 3:**
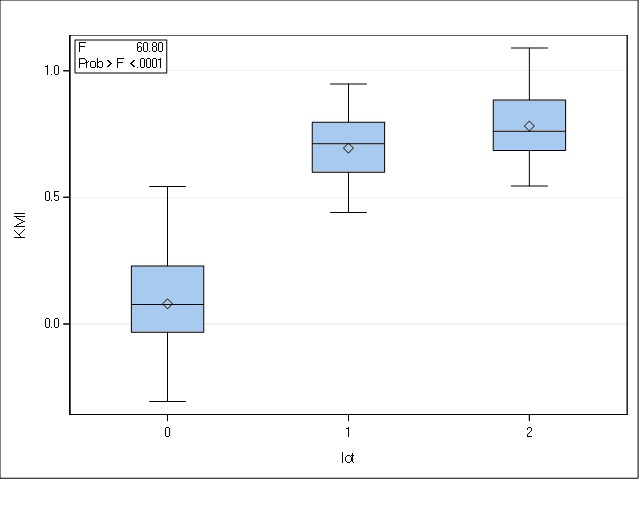
Distributions of KMI in the three groups (0 = keratoconus group; 1 = family members, 2 = controls)

The result of the test (F = 60.80, gl = (2, 39), p < 0.01) allowed us to state that there were significant differences between the three sets of participants, verifying it with the Tukey procedure. We found differences with statistical significance between the keratoconus group and their first-degree family members, respectively between the keratoconus group and the controls.

**Table 3:** Statistical analysis of the mean differences using the Tukey test

Contrast KMI	Mean differences	Adjusted Tukey p value	CI95%
0 vs. 1	-0.61	< 0.0001	-0.78 to -0.44
0 vs. 2	-0.70	< 0.0001	-0.87 to -0.52
1 vs. 2	-0.08	0.4937	-0.27 to 0.09

## Discussions

Taking into account the higher risk of developing keratoconus in relatives, we compared corneal hysteresis, corneal resistance factor and keratoconus match index in patients with keratoconus with their first-degree family members and a control group and observed lower values in the relatives when compared with the controls. These results were in accordance with the study conducted by Kara et al., which stated that these biomechanical properties could be used in assessing a subject at risk, suggesting that these may detect early changes of a diseased cornea even before the topographic indices [**31**]. The findings highlighted the altered viscoelastic capacities in keratoconic corneas as well as in the group of relatives.

Furthermore, Schwitzer et al. evidenced the finding of the lower values of CH and CRF in forme fruste keratoconus compared with healthy corneas with the hope of detecting subclinical keratoconus [**32**]. Transposing this hypothesis into our study, the group of relatives presented decreased values of CH, CRF, and KMI compared with the controls, indicating that even though the topographic indices were inside normal limits, the corneal biomechanical properties may be affected in some degrees. Many studies evaluated the dynamics of biomechanical properties after corneal collagen crosslinking (CXL) using ORA and observed an improvement of some parameters after the intervention but no significant changes in CH or CRF long-term after CXL [**33**-**35**].

## Conclusions

The screening in keratoconus remains a clinical challenge, especially in the case of first-degree family members of the keratoconus patients who are at higher risk of developing the disease. These subjects should be closely and regularly assessed not only by corneal topography but also by Ocular Response Analyzer, since even a discretely modified biomechanical parameter could be an indicator of corneal sufferance and implicitly an early premonitory sign of a subclinical keratoconus. 
